# Estimated Childhood Lead Exposure From Drinking Water in Chicago

**DOI:** 10.1001/jamapediatrics.2024.0133

**Published:** 2024-03-18

**Authors:** Benjamin Q. Huynh, Elizabeth T. Chin, Mathew V. Kiang

**Affiliations:** 1 Department of Environmental Health and Engineering, Johns Hopkins Bloomberg School of Public Health, Baltimore, Maryland; 2 Department of Biostatistics, Johns Hopkins Bloomberg School of Public Health, Baltimore, Maryland; 3 Department of Epidemiology & Population Health, Stanford University School of Medicine, Stanford, California

## Abstract

**Importance:**

There is no level of lead in drinking water considered to be safe, yet lead service lines are still commonly used in water systems across the US.

**Objective:**

To identify the extent of lead-contaminated drinking water in Chicago, Illinois, and model its impact on children younger than 6 years.

**Design, Setting, and Participants:**

For this cross-sectional study, a retrospective assessment was performed of lead exposure based on household tests collected from January 2016 to September 2023. Tests were obtained from households in Chicago that registered for a free self-administered testing service for lead exposure. Machine learning and microsimulation were used to estimate citywide childhood lead exposure.

**Exposure:**

Lead-contaminated drinking water, measured in parts per billion.

**Main Outcomes and Measures:**

Number of children younger than 6 years exposed to lead-contaminated water.

**Results:**

A total of 38 385 household lead tests were collected. An estimated 68% (95% uncertainty interval, 66%-69%) of children younger than 6 years were exposed to lead-contaminated water, corresponding to 129 000 children (95% uncertainty interval, 128 000-131 000 children). Ten-percentage-point increases in block-level Black and Hispanic populations were associated with 3% (95% CI, 2%-3%) and 6% (95% CI, 5%-7%) decreases in odds of being tested for lead and 4% (95% CI, 3%-6%) and 11% (95% CI, 10%-13%) increases in having lead-contaminated drinking water, respectively.

**Conclusions and Relevance:**

These findings indicate that childhood lead exposure is widespread in Chicago, and racial inequities are present in both testing rates and exposure levels. Machine learning may assist in preliminary screening for lead exposure, and efforts to remediate the effects of environmental racism should involve improving outreach for and access to lead testing services.

## Key Points

**Question:**

What is the extent and impact of lead-contaminated drinking water in Chicago, Illinois?

**Findings:**

In this cross-sectional study, an estimated 68% of children younger than 6 years in Chicago are exposed to lead-contaminated drinking water, with 19% of affected children using unfiltered tap water as their primary drinking water source. Predominantly Black and Hispanic blocks were disproportionately less likely to be tested for lead yet disproportionately exposed to contaminated drinking water.

**Meaning:**

Childhood lead exposure from drinking water is widespread in Chicago, with racial inequities in both testing rates and exposure levels.

## Teaser

This cross-sectional study evaluates the extent and impact of lead-contaminated drinking water in children younger than 6 years in Chicago, Illinois.

## Introduction

Lead exposure has been known to pose significant health risks, leading to historically successful large-scale efforts to reduce lead exposure from common sources, such as gasoline or paint, in the US. However, lead contamination in drinking water remains a public health concern that has garnered heightened attention in recent years.^1^ Lead pipes, which were commonly used in water systems until their federal ban in 1986, can leach lead into the water supply, especially when the water contains corrosive materials.^1,2^ There is no level of lead in drinking water considered to be safe for consumption, with the Environmental Protection Agency setting the maximum contaminant level goal for lead in water at 0.^3^ Even low-level exposure to lead-contaminated drinking water has been found to be associated with increased blood lead levels (BLLs), and even modestly elevated BLLs are associated with adverse outcomes.^4,5,6,7,8,9,10,11^ Lead exposure can have serious health consequences, particularly for children, including developmental deficits, cardiovascular complications, chronic kidney disease, and neurologic complications.^1,2,5,12,13^

Many cities across the US still have lead service lines in their water systems, including Chicago, Illinois, where lead pipes were mandated until the 1986 federal ban.^14^ Chicago is estimated to have nearly 400 000 lead service lines (where 1 line serves approximately 1 household), the most of any US city.^15^ Despite efforts to identify and replace lead service lines, progress has been slow, with only 280 (0.007%) lead pipes replaced by the city government from 2020 to 2022. To collect data on lead-contaminated water, the city offers free self-testing kits for residents. However, such a data collection approach may be subject to selection bias, potentially obscuring the true prevalence of lead exposure.

This cross-sectional retrospective study examined the extent and impact of childhood lead exposure across Chicago on the basis of household tests. Machine learning, regression, and microsimulation models were used to estimate the prevalence of childhood lead exposure from drinking water, identify racial inequities in terms of lead prevalence and screening, and model potential BLL increases in children exposed to lead-contaminated drinking water.

## Methods

This cross-sectional study did not require review based on the Johns Hopkins Institutional Review Board process, as it does not meet the criteria for human participant research. Specifically, this study was a secondary data analysis of deidentified, delinked data, and investigators had no role in its original collection. Informed consent does not apply because this was not human participant research. The Strengthening the Reporting of Observational Studies in Epidemiology (STROBE) guideline was followed in the design of this study. The eMethods in Supplement 1 provide more information on all aspects of the study setting, models, and analyses.

### Data Sources

We used publicly available lead testing data provided by the Chicago Department of Water Management containing 38 385 lead tests collected from January 2016 to September 2023. Tests were partially anonymized with the last 2 digits of each address truncated; the tests encompassed 12 139 census blocks and at least 14 673 unique addresses.^16^ Each test contained at least 3 distinct measurements of lead concentrations: a first draw after water had been kept stagnant for at least 6 hours, a second draw after 2 minutes of flushing, and a third draw after 5 minutes of flushing.

We obtained sociodemographic information using 5-year estimates from the 2021 American Community Survey^17^ and the 2020 Census,^18^ including information such as population, race, education, poverty, building age, and home value. Race and ethnicity were categorized as Asian, Black, Hispanic, and White. We also used data from the Chicago Health Atlas^19^ for tract-level health metrics, and data on building footprints were obtained from the City of Chicago.^20^ We obtained citywide survey responses to the Healthy Chicago Survey in aggregate for years 2021 to 2022 from the Chicago Department of Public Health, where respondents were asked whether their primary source of drinking water was from unfiltered tap water, filtered tap water, bottled water, or some other source, aggregated by neighborhood. Our data set consisted of 33 786 residential census blocks in total, selected by filtering for blocks with nonzero block populations within Chicago. We assessed the characteristics of census blocks in our study by aggregating over block-level information, such as population by race and ethnic group and building features. An overview of our data sources and analytical strategy is in eFigure 1 and eTable 1 in Supplement 1.

### Estimating Risk of Lead Exposure

We trained machine learning models to determine the block-level risk of having lead-contaminated drinking water, defined as the majority (≥50%) of tests within a block having 1 part per billion (ppb) or more of lead concentration in the second draw. The value of 1 ppb was chosen as the decision threshold because (1) no amount of lead in drinking water is considered safe for consumption, and (2) 1 ppb is the limit of detection for the lead water tests, making it a natural threshold for determining whether a household has lead-contaminated drinking water. We chose the second draw as an outcome because it had the highest median lead concentration, suiting our intent to identify households with lead-contaminated drinking water.

We tuned our models via cross-validation on a training set using LightGBM,^21^ a gradient-boosting decision tree algorithm, as our final model and used block-level sociodemographic and building age variables as predictors (eTable 1 in Supplement 1). Model performance was evaluated on the basis of predictions on a held-out data set that was never used for model training. As robustness checks, we (1) trained models using tests as the unit of observation instead of census blocks and (2) trained models using the first and third draws instead of the second draw. We computed model explanations using Shapley Additive Explanations, a method to interpret the output of machine learning models by decomposing predictions into additive feature contributions.^22,23^

### Assessing Disparities in Lead Screening and Exposure

To assess racial disparities in lead screening, we conducted risk-adjusted logistic regressions,^24^ a method leveraging machine learning output to measure disparities while adjusting for omitted-variable and included-variable bias. The outcome was whether a census block was screened, and covariates included the proportion of block-level population by racial and ethnic group and predictions of estimated risk of lead exposure from our machine learning model. We included estimated risk as a covariate to explicitly assess whether testing disparities exist even after adjusting for risk of lead exposure. As a robustness check, we conducted regressions without adjusting for estimated risk.

To assess disparities in lead exposure, we conducted logistic regressions with lead exposure as a binary outcome and with block-level population by racial and ethnic group and block-level population overall as covariates. Regressions were conducted separately for each racial and ethnic group. For all regressions, we calculated E-values, which indicate the minimum strength an unobserved confounder would need to have to explain away the association identified from the regression using the calculation method for odds ratios with common outcomes.^25^ As a robustness check, we conducted exposure odds analyses using tests as the unit of observation instead of census blocks.

### Estimating Extent of Childhood Exposure to Lead-Contaminated Drinking Water

To estimate the extent of childhood exposure to lead-contaminated drinking water per block, we used a Monte Carlo microsimulation approach with 10 000 simulations. To model block-level child population younger than 6 years, we used the children younger than 5 years and children younger than 10 years variables from the American Community Survey; we calculated the number of 5-year-old children as the number of 5- to 9-year-old children divided by 5, assuming children were equally distributed across years of age. We classified each block as having either lead exposure or no lead exposure based on output from our machine learning models and adjusted for misclassifications by sampling from a binomial distribution based on the known positive and negative predictive values of our machine learning model. We then determined number of children exposed by summing the number of children over all blocks modeled as having lead exposure; we multiplied these estimates by block-level measurements of race and ethnicity and reported primary source of drinking water to obtain stratified estimates. For blocks modeled as having nonzero lead exposure, we modeled drinking water lead concentration by aggregating all test results with nonzero lead concentration from their respective geographic regions and sampled from them with replacement.

We calculated the relative increase of BLL attributable to lead-contaminated drinking water for blocks modeled as having lead exposure by using an identified exposure-response association between lead-contaminated drinking water and BLL in children aged 1 to 5 years in Montreal, Quebec, Canada, after 150 days of exposure.^11^ To do this, we sampled from a uniform distribution centered on their estimate for the increase in BLL per ppb of lead in drinking water, bounded by the 95% CIs of the estimate. Because their estimates were based on lead-contaminated water samples that did not exceed 10 ppb, we conservatively top-coded all concentration observations above 10 ppb to be 10 ppb (10% of observations were above 10 ppb). We chose their most conservative specification for the effect of lead in water on BLL, using adjustments for other forms of lead exposure. As robustness checks, we ran our microsimulations using the unadjusted exposure-response association between lead water concentration and BLL using probabilistic estimations and using household lead tests as the observation unit instead of census blocks. All parenthetical measures of uncertainty for results from our simulations are reported in 95% uncertainty intervals, or the interval between the 5th and 95th percentiles of values over all simulations.

### Statistical Analysis

All the analyses were performed with the use of R software, version 3.6.3 (R Project for Statistical Computing). Additional details are provided in Supplement 1.

## Results

### Population Characteristics

Our lead testing data set included 38 385 household lead tests. Census blocks with testing results encompassed 36% of 33 786 residential census blocks. Tested and untested blocks had different racial, ethnic, and geographic distributions (Table 1 and Figure) but similar median building ages. Sixty-nine percent of lead tests yielded 1 ppb or greater lead concentration, and 33% of lead tests yielded 5 ppb or greater lead concentration (Table 2). Among 8360 citywide survey respondents, 1859 (weighted percentage, 20%) used unfiltered tap water as their primary source of drinking water (eFigure 2 and eTable 2 in Supplement 1).

**Table 1.** Characteristics of Chicago Census Blocks, Stratified by Lead Testing Status

Variable	Tested	Untested	Total
Tests, No.	38 385	NA	38 385
Census blocks, No.	12 139	21 647	33 786
Buildings per block, median (IQR), No.	18 (14-26)	15 (11-19)	16 (13-20)
Building age, median (IQR), y	97 (68-112)	98 (70-114)	97 (69-114)
Block population, median (IQR), No. of residents	76 (44-110)	53 (31-85)	58 (35-94)
Race and ethnicity, No. (%) of individuals			
Asian	404 174 (8.60)	97 049 (6.40)	192 538 (7.00)
Black	907 782 (19.30)	483 658 (31.90)	801 161 (29.20)
Hispanic	914 672 (19.50)	512 185 (33.70)	819 433 (29.80)
White	2 471 802 (52.70)	462 602 (30.50)	985 820 (35.90)

Abbreviation: NA, not applicable.

**Figure.**
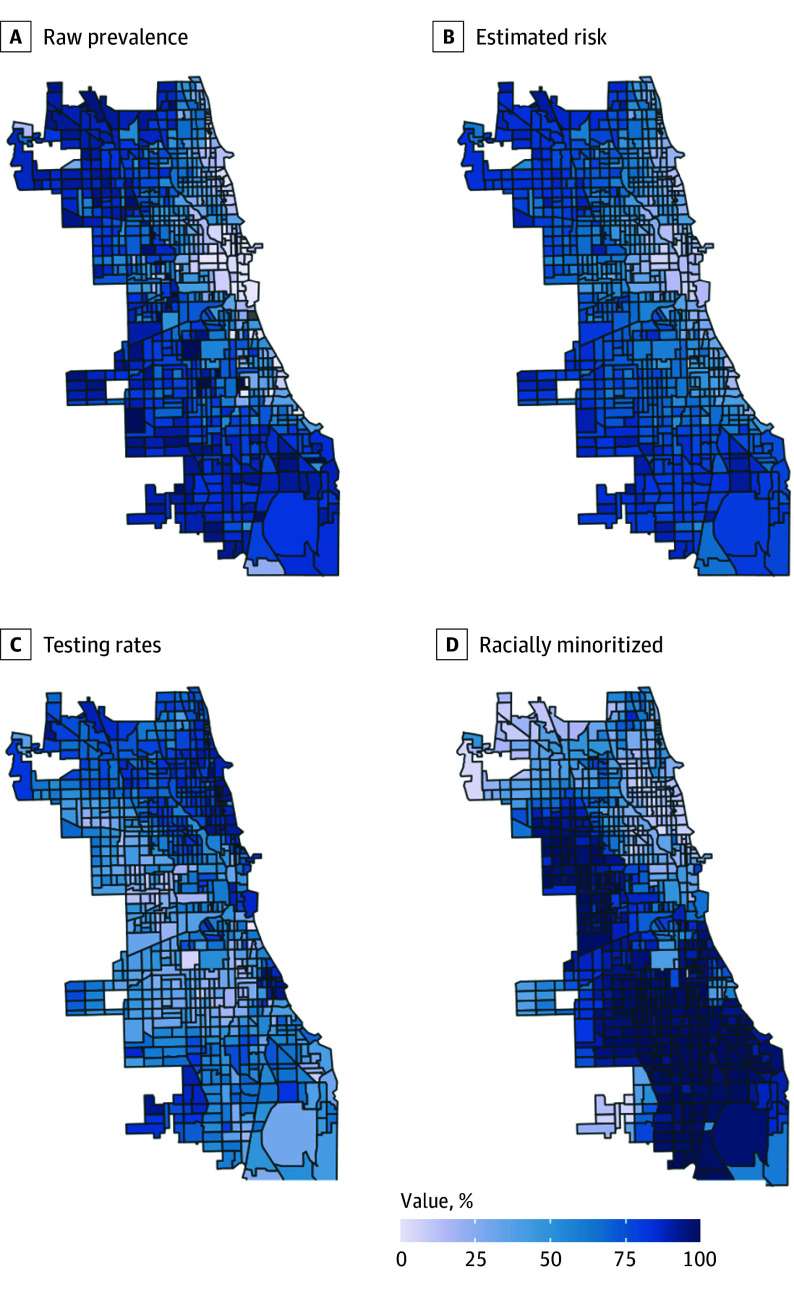
Census Tract–Level Maps of Chicago, Illinois, With Indicators of Lead Exposure and Screening A, Raw prevalence of lead exposure based on available data. B, Estimated risk of lead exposure from machine learning model. C, Rates of testing for lead exposure. D, Percentages of each tract populated by racially minoritized individuals.

**Table 2.** Results of Lead Water Testing in Chicago, Illinois

Variable	ppb, Median (IQR)	No. (%)		
		≥1 ppb	≥5 ppb	≥15 ppb
First draw	2.3 (4.6)	27 481 (71.59)	8750 (22.8)	1710 (4.45)
2-min Flush	2.6 (6.7)	26 379 (68.72)	12 670 (33.01)	3339 (8.7)
5-min Flush	1.5 (3.1)	23 552 (61.36)	5021 (13.08)	838 (2.18)

Abbreviation: ppb, parts per billion.

### Lead Exposure Risk Estimates

The machine learning model was able to predict lead exposure on a held-out test set with an area under the receiver operating characteristic curve of 0.81 (eTable 3 in Supplement 1). A total of 75% of all census blocks were estimated to have lead-contaminated drinking water (eTable 4 in Supplement 1). The top predictors were geographic units (community area, census tract, and census block group), block population, and number of buildings per block (eFigure 3 in Supplement 1). Blocks with larger populations had lower estimated risk, and blocks with a higher number of buildings per block had higher estimated risk.

### Disparities in Screening Rates and Lead Exposure

Racial disparities in screening rates and lead exposure were present. Ten-percentage-point increases in block-level Black and Hispanic populations were associated with 3% (95% CI, 2%-3%) and 6% (95% CI, 5%-7%) decreases in screening odds, respectively; a 10% increase in White population was associated with a 24% (95% CI, 23%-25%) increase in screening odds. Concurrently, 10-percentage-point increases in Black and Hispanic populations were associated with 4% (95% CI, 3%-6%) and 11% (95% CI, 10%-13%) increases in lead exposure, respectively; a 10-percentage-point increase in block-level White population was associated with a 5% (95% CI, 4%-6%) decrease (Table 3; eFigure 4 and eTable 5 in Supplement 1). Black and Hispanic survey respondents reported the lowest rates of unfiltered tap water use (weighted percentages, 14% and 12%, respectively), and White respondents reported the highest (weighted percentage, 32%) (eTable 2 in Supplement 1).

**Table 3.** Regression Results for Identifying Racial Disparities in Lead Screening and Exposure^a^

Racial group	Screening odds					Lead exposure odds				
	Coefficient	SE	Increase (95% CI)	*P* value	E-value	Coefficient	SE	Increase (95% CI)	*P* value	E-value
Asian	0.85	0.11	13.41 (10.99 to 15.83)	<.001	2.43	−1.88	0.19	−8.48 (−12.98 to −3.98)	<.001	4.57
Black	−0.29	0.03	−2.51 (−3.11 to −1.91)	<.001	1.58	0.37	0.05	4.44 (3.33 to 5.55)	<.001	1.69
Hispanic	−0.97	0.04	−6.21 (−7.03 to −5.4)	<.001	2.63	0.75	0.07	11.21 (9.66 to 12.77)	<.001	2.27
White	1.23	0.04	24.27 (23.46 to 25.07)	<.001	3.11	−0.69	0.06	−4.98 (−6.31 to −3.64)	<.001	2.17

^a^ Regressions were conducted separately for each race, and race is measured by percentage composition per block. Increase is interpreted as the percentage increase in screening or exposure odds per 10-percentage-point increase in racial group population. Coefficient refers to the regression coefficient, and SE refers to its SE; E-value is the minimum strength of association (on a risk ratio scale) a confounder would need to explain away the association.

### Childhood Lead Exposure Estimates

Our machine learning model estimated 75% of 33 786 residential census blocks to have lead-contaminated water (Figure). A total of 129 000 of all children younger than 6 years (68%; 95% uncertainty interval, 66%-69%) were estimated to be exposed to lead-contaminated water. An estimated 19% of exposed children (n = 22 400) used unfiltered tap water as their primary drinking water source, corresponding to an estimated 103% (95% uncertainty interval, 46.7%-162%) increase in BLLs after 150 days of exposure (Table 4 and eTables 6-8 in Supplement 1).

**Table 4.** Estimated Lead Exposure and Relative BLL Increase Attributable to Lead-Contaminated Drinking Water Among Children Younger Than 6 Years, Stratified by Race^a^

Racial group	No. (95% uncertainty interval)			Mean (95% uncertainty interval)		
	Child population	Affected children	Affected children using unfiltered tap water	% BLL increase among affected	% BLL increase among overall population
Total	191 000 (190 000-193 000)	129 000 (128 000-131 000)	24 400 (22 600-26 100)	103 (46.7-162)	13.1 (5.9-20.7)
Asian	11 800 (11 600-12 000)	6970 (6720-7250)	1610 (1430-1800)	102 (46.3-160)	13.9 (6.3-22.4)
Black	58 900 (58 000-59 900)	39 200 (38 200-40 200)	5680 (4950-6420)	106 (47.8-166)	10.1 (4.6-16.3)
Hispanic	61 200 (60 600-61 800)	47 200 (46 500-47 900)	8220 (7350-9090)	100 (45.4-157)	13.4 (6.1-21.3)
White	63 700 (63 000-64 400)	40 100 (39 300-40 800)	9440 (8780-10 100)	104 (47.1-163)	15.4 (7.0-24.3)

Abbreviation: BLL, blood lead level.

^a^ The 95% uncertainty interval indicates the interval between the 5th and 95th percentiles of simulation results.

## Discussion

In this cross-sectional retrospective study, childhood exposure to lead-contaminated drinking water was estimated to be widespread in Chicago. We estimated that more than two-thirds of children are exposed to lead-contaminated drinking water, and among those exposed, 19% use unfiltered tap water as their main source of drinking water. We observed from our models that long-term consumption of contaminated water could lead to substantial increases in BLLs.

Increased BLLs in children can cause deficits in cognitive development and other adverse health outcomes.^4,5,6,7,12,13,26^ The impact of low-level, long-term exposure to lead-contaminated drinking water may not be easily identifiable at the individual level. Instead, it could cause population-level increases in adverse health outcomes, such as lower population-level mean IQ or increased preterm births, underscoring the need for reduced exposure to lead-contaminated drinking water.^5,12,13,26^

Our findings on primary drinking water sources by race and ethnic group corroborate prior findings that Black and Hispanic households disproportionately drink bottled water, and White households disproportionately drink tap water.^27^ However, bottled water is not necessarily less lead contaminated than tap water; the US Food and Drug Administration sets the lead concentration limit in bottled water to 5 ppb. Similarly, using filtered tap water does not necessarily prevent lead exposure, as many consumer-grade filters do not remove lead, and some households may not change their filters as consistently as is required. The extent to which groups primarily using filtered tap water or bottled water are exposed to lead-contaminated drinking water is therefore unknown.

The racial and ethnic disparities present are indicative of the myriad ways environmental racism can manifest. Lower screening rates, lower consumption of tap water, and higher levels of lead exposure among predominantly Black and Hispanic blocks may indicate mistrust toward water sources or lack of community engagement from relevant authorities.^27,28,29,30^ Neighborhoods with high-risk estimates as well as low screening rates were largely clustered in the south and west sides of the city, corresponding to the city’s geographic history of segregation and disinvestment (Figure).^7,31,32,33^

This study contributes to the existing literature by estimating population-level exposure to and relative BLL increase attributable to lead-contaminated drinking water. Prior studies have simulated impacts of all-source lead exposure and identified associations between lead-contaminated drinking water and BLLs, the results of which enabled our modeling approach.^6,11,12,13^ Other studies have used machine learning to detect lead service lines^34^ or determine lead poisoning rates^35,36^; one of these studies, conducted in Chicago, achieved a validated area under the receiver operating characteristic curve of 0.69 in estimating elevated BLLs in children.^35^ Our study extends the literature by using machine learning models to estimate out-of-sample population-level prevalence of lead exposure and model population-level impacts of exposure.

### Limitations

Some limitations to our study include data resolution and missingness. Lead contamination levels were partially anonymized by truncating the last 2 digits of each address, so we were unable to incorporate household-specific data; an analysis done on the deanonymized data set would likely have stronger predictive performance. Second, we used machine learning to estimate the risk of lead contamination for households that did not test for lead, but this approach assumes the relationship between variables and lead exposure to be the same between the observable data and the out-of-sample data; actual data from those households may vary, and equitable screening and data collection for all at-risk neighborhoods should remain a priority to reduce lead exposure. Third, we do not have representative data on BLLs across the city, meaning we are only able to estimate relative increases in BLL. This lack of data in combination with lack of sufficiently identified dose-response associations led us to not model many health outcomes from lead exposure, such as IQ loss, maternal health, cardiovascular outcomes, or developmental disorders.^4,5,6,7,12,13,26^ For example, low-level lead exposure has been shown to be associated with increased preterm births,^26^ but we are unable to model these outcomes due to lack of (1) representative information on baseline BLLs and (2) a suitable exposure-response function for lead-contaminated drinking water and BLLs among pregnant women.

Other limitations involve modeling assumptions. Our model estimates of relative BLL increase are based on a Montreal study on children aged 1 to 5 years and assume generalizability of the exposure-response function to children in Chicago. The geometric mean of BLL in the Montreal study was 13.4 μg/L (95% CI, 5.0-36.1 μg/L). The mean BLL in Chicago is not known, but the reported Illinois geometric mean in 2019 was 21 μg/L,^37^ and the reported national geometric mean was 8.3 μg/L (95% CI, 7.8-8.8 μg/L) from 2011 to 2016,^38^ neither of which corresponds exactly to the Montreal mean but do fall within its CIs. The mean age of children from the Montreal study is 41 months; our modeled child population assumes an even distribution across years of age, with a mean age of 42 months. To account for these potential population differences, we presented our estimates of BLL increases across a wide interval of possible values to represent model uncertainty. Our model also assumes children in Chicago have similar water consumption habits and age distributions as children from the Montreal study. Our estimates of relative BLL increase only apply to children aged 1 to 5 years, as infants up to 12 months old were excluded from the Montreal study and consume more water relative to their body weight. Similarly, our estimates of BLL increases cannot be extrapolated beyond 150 days, as the original study only calculated lead exposure to 150 days preceding BLL measurements.^39^

A last category of study limitations is that our study does not investigate sources of lead exposure beyond drinking water. Racial and ethnic disparities in elevated BLLs in children may be driven by other environmental factors, such as dust and paint for Black and Hispanic children and imported food for Asian children.^33,40,41,42,43,44^ Efforts to reduce childhood lead exposure should take a holistic view across different environmental factors and sociocultural contexts.

## Conclusion

Levels of widespread childhood lead exposure, such as those found in this study, are symptomatic of structural marginalization and are likely preventable through large-scale interventions to replace lead service lines and improve access to testing. The benefits of harm-reduction strategies, such as lead filtration technology and anticorrosive agents to prevent lead leaching into water, should also be studied and explored. Machine learning may be useful as a preliminary screening tool, and a holistic approach to supplement data-driven identification with community-based input could help prevent lead exposure. Further action should be taken to reduce childhood lead exposure from drinking water.

## Notes

Supplement 1.
eMethods.
**eFigure 1.** Overview of Data Sources and Study Design **eFigure 2.** Unfiltered Tap Water Usage as Primary Drinking Water Source by Community Area in Chicago, as Reported by the Healthy Chicago Survey From Years 2021 and 2022 **eFigure 3.** Variable Explanation Visualizations Using Shapley Additive Explanations (SHAP) for a Machine Learning Model Predicting Risk of Lead Exposure **eFigure 4.** Distribution of Block-Level Observations as They Relate to Percent Race/Ethnicity and Estimated Risk of Lead Exposure, Derived From Our Machine Learning Models **eTable 1.** List of Predictor Variables Used for Machine Learning Models **eTable 2.** Survey Results for the Healthy Chicago Survey Indicating Respondents' Primary Source of Drinking Water **eTable 3.** Machine Learning Performance Results Under Different Machine Learning Algorithms **eTable 4.** Prevalence Estimates for Lead-Contaminated Drinking Water in Chicago, Stratified by Census Blocks With and Without Tests **eTable 5.** Regression Results for Identifying Racial Disparities in (A) Lead Screening and (B) Exposure **eTable 6.** Estimated Lead Exposure and Relative Blood Lead Level Increase Attributable to Lead-Contaminated Drinking Water Among Children Under 6-Years-Old, Stratified by Race; Uses Unadjusted Exposure-Response Relationship **eTable 7.** Estimated Lead Exposure and Relative Blood Lead Level Increase Attributable to Lead-Contaminated Drinking Water Among Children Under 6-Years-Old, Stratified by Race; Uses Probabilistic Predictions **eTable 8.** Estimated Lead Exposure and Relative Blood Lead Level Increase Attributable to Lead-Contaminated Drinking Water Among Children Under 6-Years-Old, Stratified by Race; the Underlying Machine Learning Model Uses Tests as the Unit of Observation Instead of Census Blocks

Supplement 2.
Data Sharing Statement
