# Supplementary material for: Estimated Childhood Lead Exposure From Drinking Water in Chicago
Source: JAMA Pediatr. 2024 Mar 18;178(5):473–9. doi: 10.1001/jamapediatrics.2024.0133 (PMC10949143; doi:10.1001/jamapediatrics.2024.0133)
Supplement: Supplement 1. — eMethods. eFigure 1. Overview of Data Sources and Study Design eFigure 2. Unfiltered Tap Water Usage as Primary Drinking Water Source by Community Area in Chicago, as Reported by the Healthy Chicago Survey From Years 2021 and 2022 eFigure 3. Variable Explanation Visualizations Using Shapley Additive Explanations (SHAP) for a Machine Learning Model Predicting Risk of Lead Exposure eFigure 4. Distribution of Block-Level Observations as They Relate to Percent Race/Ethnicity and Estimated Risk of Lead Exposure, Derived From Our Machine Learning Models eTable 1. List of Predictor Variables Used for Machine Learning Models eTable 2. Survey Results for the Healthy Chicago Survey Indicating Respondents' Primary Source of Drinking Water eTable 3. Machine Learning Performance Results Under Different Machine Learning Algorithms eTable 4. Prevalence Estimates for Lead-Contaminated Drinking Water in Chicago, Stratified by Census Blocks With and Without Tests eTable 5. Regression Results for Identifying Racial Disparities in (A) Lead Screening and (B) Exposure eTable 6. Estimated Lead Exposure and Relative Blood Lead Level Increase Attributable to Lead-Contaminated Drinking Water Among Children Under 6-Years-Old, Stratified by Race; Uses Unadjusted Exposure-Response Relationship eTable 7. Estimated Lead Exposure and Relative Blood Lead Level Increase Attributable to Lead-Contaminated Drinking Water Among Children Under 6-Years-Old, Stratified by Race; Uses Probabilistic Predictions eTable 8. Estimated Lead Exposure and Relative Blood Lead Level Increase Attributable to Lead-Contaminated Drinking Water Among Children Under 6-Years-Old, Stratified by Race; the Underlying Machine Learning Model Uses Tests as the Unit of Observation Instead of Census Blocks [file jamapediatr-e240133-s001.pdf]

Supplementary Online Content

Huynh BQ, Chin ET, Kiang MV. Estimated childhood lead exposure from drinking water in Chicago. *JAMA Pediatr*. 2024;178(3.3):e240133. doi:10.1001/jamapediatrics.2024.0133

eMethods.
eFigure 1. Overview of Data Sources and Study Design
eFigure 2. Unfiltered Tap Water Usage as Primary Drinking Water Source by Community Area in Chicago, as Reported by the Healthy Chicago Survey From Years 2021 and 2022
eFigure 3. Variable Explanation Visualizations Using Shapley Additive Explanations (SHAP) for a Machine Learning Model Predicting Risk of Lead Exposure
eFigure 4. Distribution of Block-Level Observations as They Relate to Percent Race/Ethnicity and Estimated Risk of Lead Exposure, Derived From Our Machine Learning Models
eTable 1. List of Predictor Variables Used for Machine Learning Models
eTable 2. Survey Results for the Healthy Chicago Survey Indicating Respondents' Primary Source of Drinking Water
eTable 3. Machine Learning Performance Results Under Different Machine Learning Algorithms
eTable 4. Prevalence Estimates for Lead-Contaminated Drinking Water in Chicago, Stratified by Census Blocks With and Without Tests
eTable 5. Regression Results for Identifying Racial Disparities in (A) Lead Screening and (B) Exposure
eTable 6. Estimated Lead Exposure and Relative Blood Lead Level Increase Attributable to Lead-Contaminated Drinking Water Among Children Under 6-Years-Old, Stratified by Race; Uses Unadjusted Exposure-Response Relationship
eTable 7. Estimated Lead Exposure and Relative Blood Lead Level Increase Attributable to Lead-Contaminated Drinking Water Among Children Under 6-Years-Old, Stratified by Race; Uses Probabilistic Predictions
eTable 8. Estimated Lead Exposure and Relative Blood Lead Level Increase Attributable to Lead-Contaminated Drinking Water Among Children Under 6-Years-Old, Stratified by Race; the Underlying Machine Learning Model Uses Tests as the Unit of Observation Instead of Census Blocks

This supplementary material has been provided by the authors to give readers additional information about their work.

© 2024 Huynh BQ et al. *JAMA Pediatr*.

eMethods

I. Study setting & data

Missing data

Data on lead exposure were collected from the City of Chicago Department of Water Management, containing 38,385 lead tests over 12,139 Census blocks and at least 14,673 unique addresses, with addresses partially anonymized by truncating the last two digits. The original dataset contained 40,063 observations, but 349 observations did not have testing data and were excluded from analysis. 437 observations had invalid addresses and were excluded from analysis. To assign the block-anonymized addresses to Census blocks, we geocoded them using the *censusxy* package in R, which calls the Census geocoding API.[1] Because the last two digits in each address were anonymized, we performed an initial geocoding assigning all last two digits to be "30"; Chicago blocks can be anywhere from "00" to "99", but most residential addresses are between "00" and "60", so we chose "30" as a midpoint. This initial geocoding was able to assign Census blocks to all addresses in the dataset except for 452 observations. We then performed geocoding calls on the remaining observations using other digits, namely "49", "07", "50", and "00". We then manually geocoded each address using the Census geocoding web tool, identifying and fixing typographical errors in the original dataset. 169 observations were ultimately unable to be geocoded and were excluded from analysis. An additional 87 observations were removed because they were determined to not be within any City of Chicago Community Areas. Finally, an additional 636 observations were removed for having block populations of 0. Overall, 4.1% of observations (1678) were removed prior to analysis. As a conservative assumption, all lead concentration levels listed as "<1 part per billion" were assigned to be 0 parts per billion.

For the American Community Survey (ACS) data[2] at the Census block-group level, 0.5% of data had missing values, and were imputed using multiple imputation by chained equations, with predictors being other ACS block-group level variables, and random forest as the regression model. For the Chicago building footprint data, 1.6% of data had missing values, and were imputed using multiple imputation by chained equations[3], with predictors being all ACS and Census variables, and random forest as the regression model.

Lead water testing

Households in Chicago are able to have their water tested for lead for free, a service provided by the city. After a resident submits a request for a lead testing kit, the city sends them a kit with instructions. Residents are instructed to leave their water stagnant for at least 6 hours and no longer than 18 hours. Then, they are to set a faucet to cold water and fill one bottle for the first draw, wait 2 minutes, then fill another bottle, then wait 3 more minutes to fill the last bottle. Residents are also instructed to not use filters or aerators; filters are to be bypassed, not removed. No instructions were given as to the flow rate of the water. Residents are then instructed to schedule a pickup for the water bottles, and pickup must occur within four days of completing the test kit, at which point the city then sends the kit to a laboratory for testing. The laboratory limit of detection for lead-contaminated water is 1 ppb.

Determinants of lead concentration in drinking water

Our primary reasons for choosing 1 ppb as a natural threshold for determining lead concentration in drinking water were (1) no amount of lead in drinking water is considered safe for consumption, and (2) 1 ppb is the limit of detection for the tests used in our dataset. Other possible model specifications, such as choosing a higher threshold, or conducting regressions instead of classifications to predict lead concentration itself, would require not just determining whether a block has lead-contaminated drinking water, but also the extent to which the water is contaminated with lead. Such an approach would likely require information beyond our block-level, partially

anonymized dataset, such as household-level characteristics and construction history. Environmental characteristics like temperature could also influence lead concentration, which we would know for our training dataset, but not necessarily when making predictions for out-of-sample observations.

Multiple tests within Census blocks

With 38,385 lead tests encompassing 12,139 Census blocks and at least 14,673 unique addresses, it is likely that some of the tests are repeated observations from the same household. The median number of tests per Census block is 2, with an interquartile range of 3. eFigure 1 shows the distribution of tests per block.

Because test addresses are partially anonymized, it is impossible to determine the extent to which observations within a Census block are independent. Our primary analysis assumes dependence between within-block observations and uses Census blocks as the level of observation, where the outcome is defined as a binary variable indicating whether or not the majority of tests within a block have at least 1ppb lead concentration. Our secondary analysis, as robustness checks for our lead exposure regressions and machine learning models, assumes independence between within-block observations and uses tests as the level of observation, while ensuring that observations within blocks are always kept within the same training and test data splits to avoid data leakage.

Healthy Chicago Survey

The Healthy Chicago Survey is an annual, city-wide survey led by the Chicago Department of Public Health (CDPH), seeking to better understand the health of Chicago residents. Starting in 2021, the survey started asking respondents what their primary source of drinking water was, with options including unfiltered tap water, filtered tap water, bottled water, or some other source of drinking water. The results for these particular survey questions have not yet been publicly released but were shared with us by the CDPH.

The survey results from both 2021 and 2022 were shared with us in aggregate. We received aggregate data on survey responses by community area and race (eFigure 2, eTable 2). We did not have access to individual-level survey responses. Sampling for the survey was intended to be representative of the city, and responses were weighted accordingly. We did not have access to individual weights, but reported weighted percentages where applicable (eTable 2). Documentation for the methodology of the Healthy Chicago Survey is publicly available.[4]

Linking lead poisoning rates to lead concentrations in drinking water

Our machine learning algorithms use publicly available data on community area-level lead poisoning rates, indicating the percentage of children ages 1-5 with blood lead level at or above 50 micrograms per liter, with separate variables for each year from 2016-2021. The data are hosted on the Chicago Health Atlas and made available through the Chicago Department of Public Health (see eTable 1 for details).

Although our analysis involves estimating the relative increase in blood lead levels based on lead concentration in drinking water, we do not estimate the effect of lead-contaminated drinking water on lead poisoning rates for the following reasons: (1) due to lack of individual-level data, we cannot link instances of lead poisoning to specific households with known lead water concentration, nor can we account for children moving to different households, (2) the distribution of blood lead levels below 50 micrograms per liter is unknown, which is where we would expect to see much of the BLL increase from chronic low-level exposure to lead-contaminated drinking water, (3) these reported lead poisoning rates by community area are not necessarily representative of their neighborhoods, as BLL tests are not randomly sampled or uniformly required, and (4) we cannot adjust for other sources of lead exposure such as dust or paint.

II. Machine learning model specification

As described in the main text, we trained machine learning models to estimate the risk of a block having lead-contaminated drinking water. We describe here various details of specifying, training, and evaluating these machine learning models.

Predictor variables used

eTable 1 depicts a list of features used for our models. We created the Children under 5, 10, and 18 features using the American Community Survey (ACS) variables for age by sex, and aggregated over sex. We created the Chicago Building Footprint features by deriving age based on the years buildings were built, and calculating aggregate statistics within blocks.[5] The Chicago Health Atlas is a composite aggregation of data from various sources: the Economic Diversity Index and Hardship Index were derived from the ACS and calculated by Metopio; income, cognitive difficulties, disability, crowded housing, rent burdened, vacant housing, and uninsured rate were derived from the ACS; low food access was derived from the USDA's Food Access Research Atlas; fine particulate matter was derived from the Environmental Protection Agency; eviction rate was derived from the Eviction Lab at Princeton University; major crime was derived from the City of Chicago crime data portal; and the social vulnerability index was derived from the Centers for Disease Control and Prevention.[6]

Performance and evaluation

eTable 3 depicts machine learning model performance for the machine learning models, using three different machine learning algorithms: LightGBM[7], Random Forest[8], and glmnet[9]. LightGBM is a gradient boosting decision tree algorithm designed to be computationally inexpensive and efficient at handling sparse categorical features. Random forest is a classical machine learning algorithm that creates a large number of decision trees to produce an output. glmnet is a package for conducting regularized generalized linear models via maximum likelihood.

We split the data into a training dataset (75%) and a held-out test set (25%), and compared different machine learning algorithms using only the training dataset. For each algorithm, we used 3-fold cross-validation and tuned hyperparameters using the *tune* package in R[10], where 5 candidate hyperparameter sets were chosen automatically to be tuned. When conducting the test-level analysis, observations from the same Census block were grouped in the same cross-validation folds and dataset splits to prevent data leakage. For LightGBM, we used 1000 trees and set tree depth and minimal node size to be tuned. For the random forest models, we used 1000 trees and tuned mtry, the number of predictors to be randomly sampled at each split. For glmnet, we tuned the elastic net mixing parameter and regularization penalty.

All models performed similarly, suggesting insensitivity to model choice, but we chose LightGBM as our final model due to its superior computational speed (eTable 3). Using LightGBM, we performed a more comprehensive hyperparameter search on the same test set using 10-fold cross-validation with 25 candidate hyperparameter sets automatically chosen by *tune*. Having identified an optimal hyperparameter set, we trained on the entire training set using that set of hyperparameters, and used it to make predictions on the held-out dataset, with evaluation on that hold-out set as final predictive performance metrics. Models were assessed using area under the receiver operating characteristic curve and brier score.

Varying the outcome variable to first and third draws

As robustness checks for our machine learning model, we trained machine learning models using the first and third draws instead of the second draw as an outcome variable. We display results on both cross-validation and the held-out instead of just the cross-validation folds because we are not using these tests to determine the best model and are instead simply assessing how the model performs under different outcome variables.

The model performs slightly better using the first draw and moderately worse using the third draw (eTable 3). This is likely because the first draw has the highest prevalence of samples ≥ 1 ppb lead concentration (Table 2), and the model is therefore more able to determine whether or not the household has lead based on building and sociodemographic characteristics. Similarly, the third draw likely performs worse as an outcome variable because it flushes out lead that otherwise would have appeared as a positive test, and undercounts the number of observations with lead-contaminated drinking water.

Training on test-level data instead of block-level data

As a robustness check, we trained a machine learning model where the units of observation were tests instead of blocks. The test-level machine learning model performed slightly worse than the block-level model (eTable 3). This is likely because the test-level model had tests within blocks that had differing results, but only had block-level predictors and so made the same prediction for each test within blocks, increasing the uncertainty behind predictions and degrading performance slightly.

Probabilistic predictions

To obtain probabilistic predictions for every block in Chicago, we used calibration for potentially stronger probabilistic modeling compared to using raw prediction values. Because only blocks that were tested had observed exposure values, we first did a 50/50 split on the observed blocks only, training, tuning, and calibrating a LightGBM on one split, then predicting on the other, for both splits. Then we trained, tuned, and calibrated a LightGBM model on the entire dataset for which observed exposure values were available, and predicted on the data without observed exposure values. Beta regression was used for calibration[11]; doing so did not degrade predictive performance (AUC = .79 for both), and also slightly improved calibration in terms of Brier score (from 0.169 to 0.166).

III. Regression analyses

Estimating racial/ethnic disparities in screening without adjusting for estimated risk

As a robustness check, we estimated racial and ethnic disparities in screening without adjusting for estimated risk. We found that when conducting unadjusted regressions, estimated effect sizes were moderately attenuated, although similar qualitatively to the results of the adjusted regressions (eTable 5). We interpret this to mean that racial and ethnic disparities exist in terms of screening for lead, and that these disparities are heightened when comparing between blocks with similar levels of estimated risk of lead exposure.

Estimating racial/ethnic disparities in lead exposure at the test-level instead of block-level

As another robustness check, we estimated racial and ethnic disparities in lead exposure using tests as the unit of observation instead of blocks. In the test-level regressions, racial and ethnic disparities were increased but qualitatively similar compared to the block-level regressions (eTable 5). This is likely because of the increased number of observations when using tests instead of blocks, which had disproportionately higher White block populations.

IV. Microsimulation Model

Adjusting for machine learning model misclassification metrics

When determining whether blocks were to be modeled with or without lead exposure within each iteration of the microsimulation, we needed to account for the fact that our machine learning model was not a perfect classifier by adjusting for its misclassification metrics.[12–14] Based on the machine learning model's performance on the 50/50 dataset split we used to generate predictions for each block with tests, we calculated its false discovery rates (FDR) and false omission rates (FOR), also known as the complements of positive and negative predictive values. FDR indicates the probability that an observation classified as lead exposure does not actually have lead exposure, and FOR indicates the probability that an observation classified as not having lead exposure actually has lead exposure.

Using the calculated FDR and FOR, we sampled from a binomial distribution for each block. If the block had been predicted to have lead exposure, we sampled from the FDR, where a 1 would change the prediction from positive to negative, and a 0 would keep the prediction as positive. If the block had been predicted to not have lead exposure, we sampled from the FOR, where a 1 would change the prediction from negative to positive, and a 0 would keep the prediction as negative.

However, it is possible that the FDR and FOR rates are different between the tested population and the untested population, although we can only calculate misclassification metrics for the tested population. To account for how misclassification metrics may differ between the two different populations, we employed propensity score matching to find blocks in the tested population similar to blocks in the untested population. Specifically, we matched on variables for race (proportions of block group considered to be Asian, Black, Hispanic, and White), education (proportions of block group with high school diplomas and bachelor's degrees), building age, and number of buildings per block. We conducted nearest-neighbor matching with replacement, with a caliper of 0.15, indicating that every observation from the untested population was matched, but not necessarily every observation from the tested population.

Based on the subset of the tested population matched to the untested population, we calculated the FDR and FOR, hereafter referred to as the matched FDR and matched FOR. We then ran our Monte Carlo simulations as before, except if a block was from the untested population, we sampled using the matched FDR and matched FOR instead of the FDR and FOR. We chose this approach as our main estimate of lead exposure prevalence because it attempts to account for distributional differences between the tested and untested populations. eTable 4 shows prevalence results for each of our approaches. Prevalence estimates are similar between the tested and untested population, even though their demographic characteristics were vastly different. One possible reason is that the true prevalence may be similar between the two populations, and that differences in lead exposure are driven by building characteristics or other unobserved variables that explain away the differences in racial/ethnic distributions. Another possible reason is that our model may not have fully captured the differences between the two populations.

Modeling lead exposure per block

Chicago follows Census geographical divisions and is categorized into the following geographical units, in increasing size: block, block group, tract, and community area. Community area is a Chicago-specific designation, that is similar to what is colloquially referred to as a neighborhood, with 77 community areas in Chicago.

To model lead exposure per block, we aggregated all test results with nonzero lead concentration at the block group-level and sampled from them with replacement for each block. However, some blocks (5.3%, n = 33,786) did not have any testing data from their entire block group, so for those blocks, we sampled from their tracts instead. Some

blocks did not have any data from their entire tract or block group (2.3%, n = 33,786), so we sampled from their community areas.

Simulating sampling errors

To account for sampling error in the American Community Survey data, we varied populations and demographics based on their margins of error within each iteration of the microsimulation. Without knowledge of the true underlying distribution, propagating sampling error required distributional assumptions. We drew from uniform distributions bounded by the 95% confidence intervals of population estimates. We chose uniform distributions as a conservative representation of variability (e.g., likely overestimating variability), where extreme values in the distribution are equally likely as values close to the center. As we are only provided the point estimate and confidence intervals, specifying a distribution like the normal distribution would require making an assumption as to the underlying sample size in order to derive the standard deviation from the standard error. Margins of error for ACS variables were originally based on 90% confidence intervals, so we converted them to margins for 95% confidence intervals by dividing by the z-score corresponding to the 95th percentile of the normal distribution (~1.645) and multiplying by the z-score corresponding to the 97.5th percentile of the normal distribution (~1.96).

We used a similar approach of using a uniform distribution bounded by the reported 95% confidence intervals when simulating (1) the exposure-response function between water lead concentration and BLL increase and (2) the percentages of unfiltered tap water usage by community area. A value was randomly sampled for each iteration of the simulation. Our justification for using a uniform distribution was the same as above: we made no assumptions about the underlying sample size and instead chose a conservative modeling assumption that would likely overestimate the variability of our results.

Robustness checks

As robustness checks, we ran the microsimulation analysis multiple times with the following changes: (1) using an exposure-response relationship between lead-contaminated drinking water and BLL that was unadjusted for other sources of lead (instead of an adjusted one), (2) using probabilistic machine learning predictions for the microsimulation instead of classifications, and (3) using machine learning predictions based on a test-level model instead of a block-level model.

Using an unadjusted exposure-response relationship between lead-contaminated drinking water and increased BLL results in increased estimates of BLL increases (eTable 6). Using probabilistic machine learning predictions instead of classifications results in slightly fewer blocks being classified as having lead exposure, since the underlying prevalence of lead exposure is greater than 50% (eTable 7). Using test-level predictions instead of block-level predictions also results in fewer blocks being classified as having lead exposure, likely because having different outcomes for the same covariates (i.e., differing test results within a block) within the training set increases model uncertainty when making predictions (eTable 8).

V. Supplementary Tables and Figures

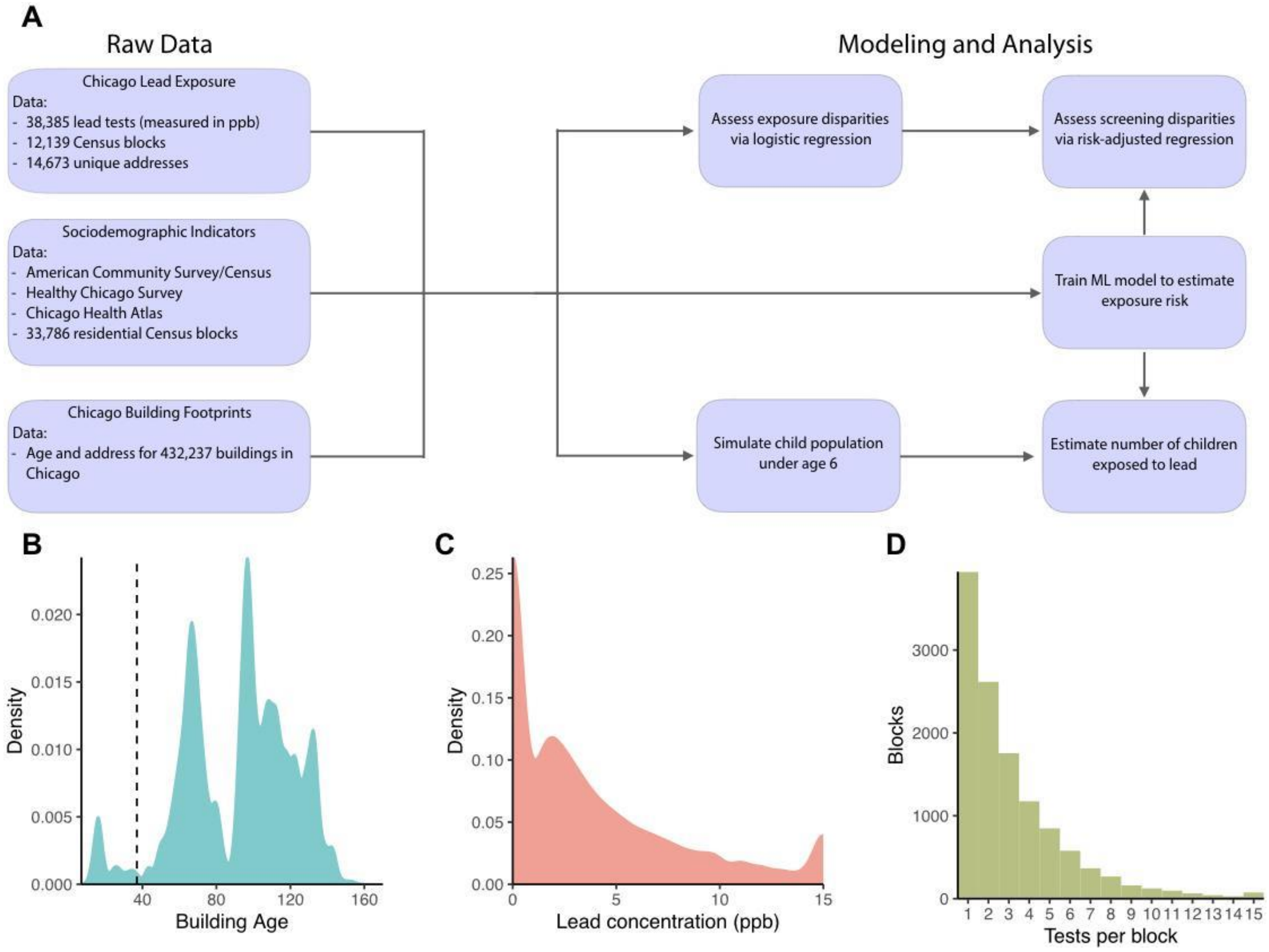

eFigure 1: Overview of data sources and study design. (A) Flow diagram of study. ML stands for machine learning. ppb indicates parts-per-billion. Neighborhood-level refers to the geographical unit known as community areas, as designated by the City of Chicago. (B) Density plot of building ages in Chicago. Dashed vertical line indicates buildings created before and after the year 1986, when lead service lines transitioned from being mandatory to prohibited for new buildings. (C) Density plot of lead sampling tests of drinking water in Chicago households. For visualization purposes only, outliers beyond 15 ppb were topcoded to 15 ppb. (D) Histogram depicting the number of lead tests per Census block among all Census blocks with at least one test. Outliers beyond 15 tests per block were topcoded to 15 for visualization purposes only.

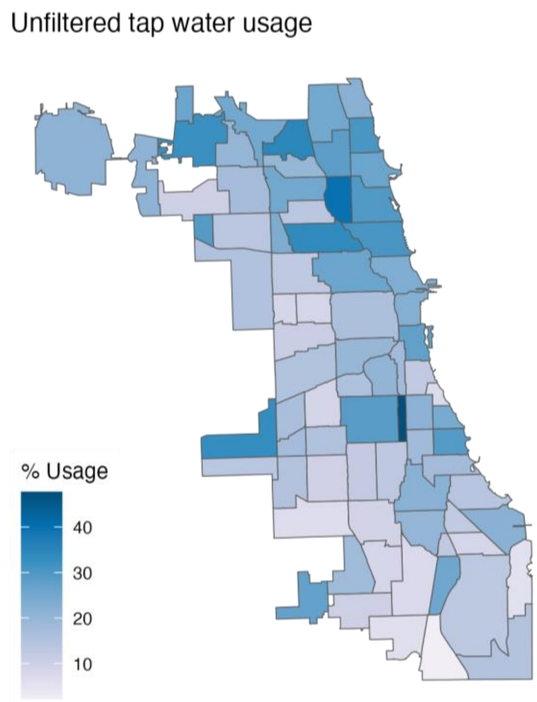

eFigure 2: Unfiltered tap water usage as primary drinking water source by Community Area in Chicago, as reported by the Healthy Chicago Survey from years 2021 and 2022.

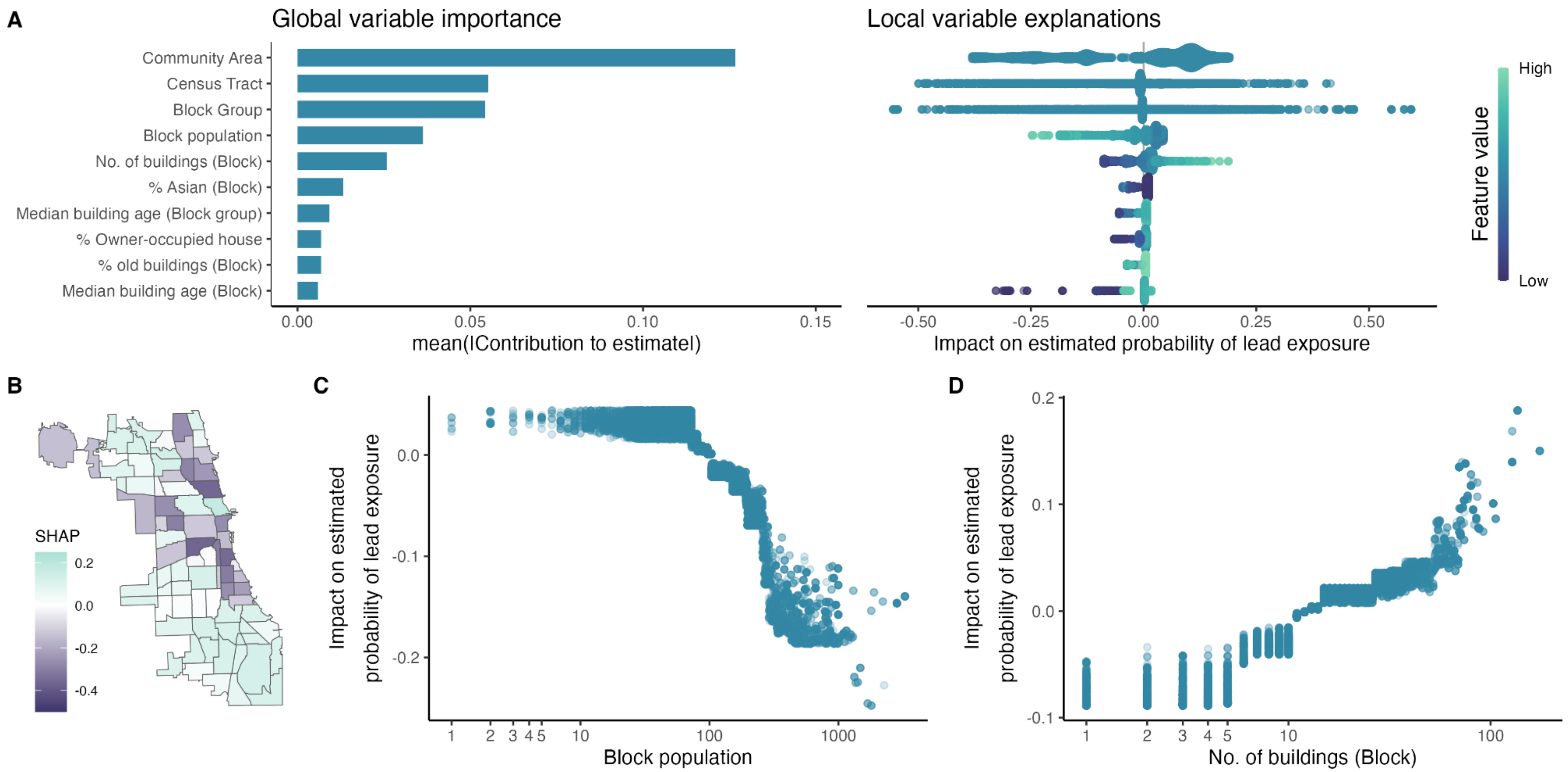

eFigure 3: Variable explanation visualizations using Shapley Additive Explanations (SHAP) for a machine learning model predicting risk of lead exposure. (A), Left: Bar plot of average SHAP magnitude, denoting variable importance. Right: Beeswarm visualization of each prediction, wherein points denote individual observations, the X-axis denotes how much a given variable influenced model output, and the color of points denotes feature values. Community area, census tract, and block group are categorical variables and therefore do not have feature values. (B), a map of community areas in Chicago, shaded by the SHAP values of categorical variables representing each community area. (C), the relationship between block population and SHAP values. (D), the relationship between number of buildings per block and SHAP values. All SHAP values depicted are normalized to indicate the impact on estimated probability of lead exposure.

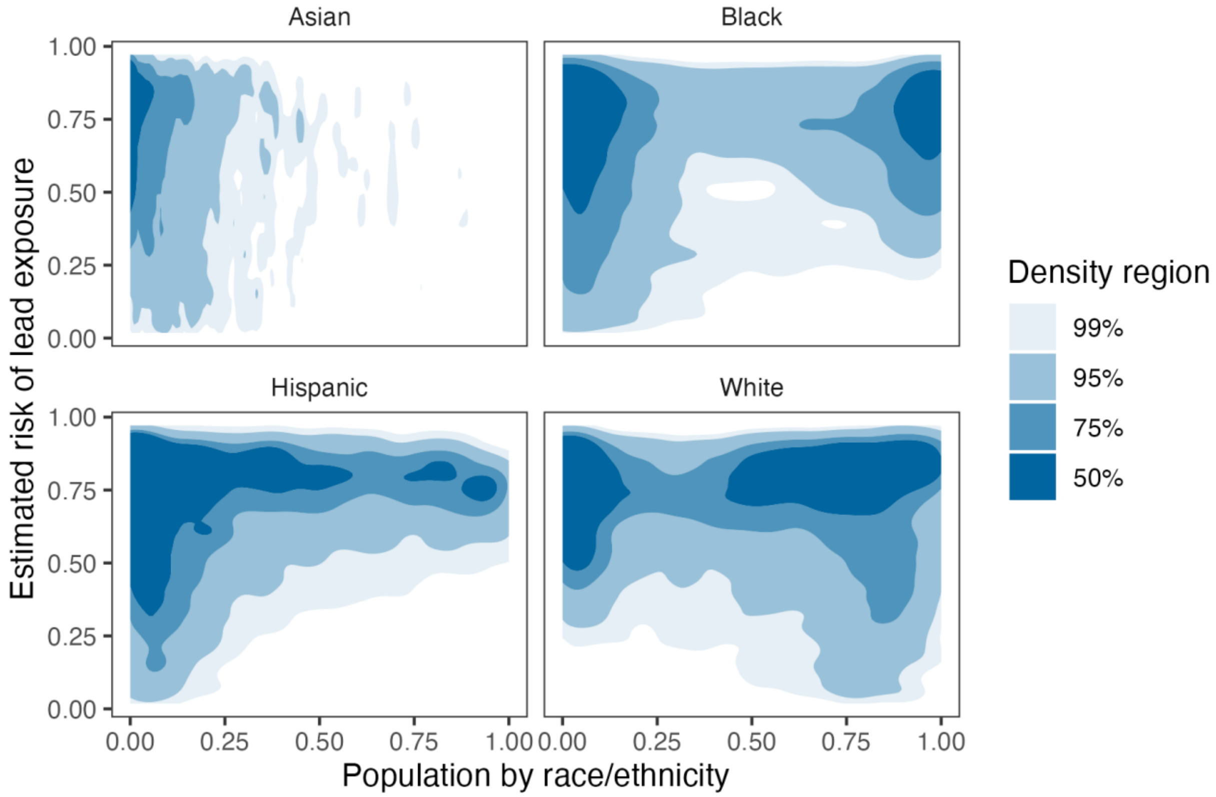

eFigure 4: Distribution of block-level observations as they relate to percent race/ethnicity and estimated risk of lead exposure, derived from our machine learning models. Density regions indicate the percentage of observations captured within that region.

eTable 1: List of predictor variables used for machine learning models.

Source	Variable	Geographic Unit(s)	Measurement
**American Community Survey[2]**	Census tract	Census tract	Identifier denoting unique census tracts
	Block number	Block	Identifier denoting unique blocks
	Block group	Block group	Identifier denoting unique block groups
	Population	Block group, census tract	Count
	Race/ethnicity	Block group, census tract	Proportion of population; groups included were American Indian/Alaskan Native, Asian, Black, Hispanic, and White
	Housing units	Block group	Count
	House value (median)	Block group	US Dollars
	House value (upper)	Block group	US Dollars
	House value (lower)	Block group	US Dollars
	Homeowner costs (median)	Block group	US Dollars
	Education	Block group	Proportion of population; levels included were high school, GED, <1 year of college, >1 year of college, Associate's Degree, Bachelor's Degree, Master's Degree, Professional School, and Doctoral Degree
	Poverty	Block group	Proportion of population
	Native born	Block group	Proportion of population
	Foreign born	Block group	Proportion of population
	English-only speakers	Block group	Proportion of population
	Speak other languages	Block group	Proportion of population
	Computer access	Block group	Proportion of households
	Internet access	Block group	Proportion of households
	Complete plumbing facilities	Block group	Proportion of households
	Vacant housing	Block group	Proportion of households
	Owner-occupied	Block group	Proportion of households
	Renter-occupied	Block group	Proportion of households
	Children under 5	Block group	Proportion of population

	Children under 10	Block group	Proportion of population
	Children under 18	Block group	Proportion of population
**Census**	Block number	Block	h
	Population	Block	
	Race/ethnicity	Block	
**Chicago Building Footprints**[5]	Median age	Block	Age
	Number of buildings	Block	Count
	Max age	Block	Age
	Mean age	Block	Age
	Built after 1986	Block	Percent
**Chicago Health Atlas**[6]	Community Area	Community area	Identifier denoting unique community areas
	Lead poisoning rate	Community area	Percent of children ages 1-5 with blood lead level at or above 50 micrograms per liter. Separate annual variables for years 2016-2021.
	Economic Diversity Index	Census tract	Probability that two randomly chosen households in a tract will belong to different multiples of the federal poverty level.
	Hardship Index	Census tract	A composite index aggregated from unemployment, age dependency, education, per capita income, crowded housing, and poverty.
	Social Vulnerability Index	Census tract	A composite index created by aggregated from 15 social factors, such as unemployment, minority status, and disability.
	Major crime	Census tract	Count
	Eviction rate	Census tract	Percent of renter-occupied households with an eviction in the past year.
	Fine particulate matter concentration	Census tract	Annual average concentration in micrograms per cubic meter.
	Access to food	Census tract	Percent of residents who are farther than 1/2 mile from the nearest supermarket in an urban area.
	Uninsured rate	Census tract	Percent of residents without health insurance.
	Income	Census tract	US Dollars
	Cognitive difficulty	Census tract	Percent of population
	Disability	Census tract	Percent of population

Crowded housing	Census tract	Percent of occupied households with more than one occupant per bedroom.
Rent burdened	Census tract	Percent of households spending more than 30% of their income on rent.
Vacant housing	Census tract	Percent of vacant households

eTable 2: Survey results for the Healthy Chicago Survey indicating respondents' primary source of drinking water.

	No. (weighted %)			
	**Unfiltered Tap Water**	**Filtered Tap Water**	**Bottled water**	**Other source**
**Total**	1859 (20.3%)	3404 (40.6%)	2926 (36.9%)	171 (2.1%)
**Asian**	95 (18.6%)	320 (57.4%)	104 (21.5%)	18 (2.6%)
**Black**	344 (14.2%)	451 (21.8%)	1390 (61%)	64 (3.1%)
**Hispanic**	215 (12.0%)	695 (41.4%)	724 (45%)	35 (1.7%)
**White**	1110 (32.1%)	1752 (51.2%)	522 (15.5%)	33 (1.2%)

eTable 3: Machine learning performance results under different machine learning algorithms. AUC refers to the area under the receiver operating characteristic curve. CV refers to the 3-fold cross-validation evaluation step during model evaluation. Hold-out refers to predictive performance on a held-out test set kept separate from the training set. First draw and third draw refer to LightGBM models trained on different datasets where the outcome is determined by lead exposure ≥ 1 ppb for the first draw (no flushing) or third draw (5 minutes of flushing) instead of the second draw (2 minutes of flushing).

Model	AUC (CV)	Std. Err	AUC (hold-out)
**LightGBM**	0.80	0.00216	0.81
**Random Forest**	0.81	0.00293	—
**glmnet**	0.80	0.00432	—
**First draw (LGBM)**	0.82	0.00352	0.83
**Third draw (LGBM)**	0.74	0.00438	0.76
**Test-level (LGBM)**	0.78	0.00582	0.79

eTable 4: Prevalence estimates for lead-contaminated drinking water in Chicago, stratified by Census blocks with and without tests. ppb indicates parts per billion. Unadjusted estimate refers to the machine learning estimate without any adjustment. Adjusted estimate refers to an adjustment to the machine learning prevalence estimate that accounts for its known positive and negative predictive values. Adjusted and matched estimate refers to an adjustment that accounts for how positive and negative predictive values may differ between the tested and untested populations.

	**Tested**	**Untested**	**Total**
**Tests ≥ 1 ppb, %**	68.7	—	68.7
**Majority of tests in block ≥ 1 ppb, %**	74.1	—	—
**Unadjusted estimate of blocks ≥ 1 ppb, %**	81.8	82.9	82.5
**Adjusted estimate of blocks ≥ 1 ppb, %**	74.1	74.7	74.4
**Adjusted and matched estimate of blocks ≥ 1 ppb, %**	74.1	75.1	74.7
**Children exposed to lead, %**	66.2	70.8	67.5
**Children exposed to lead and unfiltered tap water, %**	13.3	12.8	13

eTable 5: Regression results for identifying racial disparities in (A) lead screening and (B) exposure. Regressions were conducted separately for each race, and race is measured by percent composition per block. Increase is interpreted as the percent increase in screening/exposure odds per 10 percentage-point increase in racial group population. Coef refers to the regression coefficient, std. err refers to its standard error, p refers to the p-value, and E refers to the E-value, the minimum strength of association (on a risk ratio scale) a confounder would need to explain away the association. 95% CI refers to 95% confidence intervals. Same as Table 2, except screening odds regression is unadjusted, and exposure odds regression is conducted at the test-level instead of block-level.

	(A) Screening odds					(B) Lead exposure odds				
**Racial Group**	**Coef**	**Std. err**	**Increase (95% CI)**	**p**	**E**	**Coef**	**Std. err**	**Increase (95% CI)**	**p**	**E**
**Asian**	0.24	0.11	2.75 (0.41,5.09)	0.02	1.51	-2.85	0.11	-9.42 (-11.87,-6.97)	<0.001	7.77
**Black**	-0.25	0.03	-2.24 (-2.84,-1.65)	<0.001	1.53	0.63	0.03	8.69 (8.03,9.35)	<0.001	2.08
**Hispanic**	-0.66	0.04	-4.85 (-5.63,-4.06)	<0.001	2.13	1.02	0.05	17.82 (16.82,18.82)	<0.001	2.72
**White**	1.02	0.04	17.61 (16.84,18.39)	<0.001	2.71	-0.88	0.04	-5.86 (-6.58,-5.14)	<0.001	2.48

eTable 6: Estimated lead exposure and relative blood lead level increase attributable to lead-contaminated drinking water among children under 6-years-old, stratified by race; uses unadjusted exposure-response relationship. 95% UI refers to 95% uncertainty intervals, indicating the interval between the 5th and 95th percentiles of simulation results.Same as Table 1, but uses an unadjusted exposure-response relationship between lead-contaminated drinking water and blood lead levels.

	No. (95% uncertainty interval)			Mean, (95% uncertainty interval)	
**Racial group**	**Child Population**	**Affected Children**	**Affected Children Using Unfiltered Tap Water**	**% BLL increase among affected**	**%BLL increase among overall population**
**Total**	191000 (190000-193000)	129000 (128000-131000)	24300 (22600-26100)	155 (91.4 - 218)	19.6 (11.6 - 28)
**Asian**	11800 (11600-12000)	6960 (6720-7240)	1610 (1430-1810)	153 (91 - 216)	20.8 (12.3 - 30.3)
**Black**	58900 (58000-59900)	39200 (38200-40200)	5670 (4950-6420)	159 (93.8 - 223)	15.2 (8.88 - 22.1)
**Hispanic**	61200 (60600-61800)	47100 (46400-47900)	8210 (7360-9080)	150 (88.8 - 211)	20.1 (11.8 - 28.9)
**White**	63700 (63000-64400)	40000 (39300-40800)	9430 (8780-10100)	156 (92.3 - 220)	23 (13.7 - 32.8)

eTable 7: Estimated lead exposure and relative blood lead level increase attributable to lead-contaminated drinking water among children under 6-years-old, stratified by race; uses probabilistic predictions. 95% UI refers to 95% uncertainty intervals, indicating the interval between the 5th and 95th percentiles of simulation results. Same as Table 1, but uses probabilistic machine learning predictions instead of classifications.

	No., (95% uncertainty interval)			Mean, (95% uncertainty interval)	
**Racial group**	**Child Population**	**Affected Children**	**Affected Children Using Unfiltered Tap Water**	**% BLL increase among affected**	**%BLL increase among overall population**
**Total**	191000 (190000-193000)	123000 (121000-125000)	23400 (21800-25000)	105.0 (46.4 - 161)	12.8 (5.7 - 19.8)
**Asian**	11800 (11600-12100)	6890 (6610-7200)	1600 (1430-1780)	103.0 (45.5 - 158)	13.9 (6.2 - 21.9)
**Black**	58900 (58000-59900)	37300 (36300-38400)	5460 (4780-6140)	108.0 (47.6 - 165)	9.9 (4.4 - 15.6)
**Hispanic**	61200 (60600-61800)	42600 (41900-43300)	7500 (6750-8280)	102.0 (45.2 - 157)	12.4 (5.5 - 19.4)
**White**	63700 (63000-64400)	39300 (38500-40100)	9370 (8740-10000)	105.0 (46.6 - 162)	15.5 (6.9 - 24.0)

eTable 8: Estimated lead exposure and relative blood lead level increase attributable to lead-contaminated drinking water among children under 6-years-old, stratified by race; the underlying machine learning model uses tests as the unit of observation instead of Census blocks. 95% UI refers to 95% uncertainty intervals, indicating the interval between the 5th and 95th percentiles of simulation results. Same as Table 1, but uses tests as the unit of observation instead of Census blocks.

	**No. (95% uncertainty interval)**			**Mean, (95% uncertainty interval)**	
**Racial group**	**Child Population**	**Affected Children**	**Affected Children Using Unfiltered Tap Water**	**% BLL increase among affected**	**%BLL increase among overall population**
**Total**	191000 (190000-193000)	120000 (119000-122000)	22500 (21000-24200)	104 (47.0 - 162.0)	12.2 (5.52 - 19.2)
**Asian**	11800 (11500-12000)	6500 (6240-6780)	1500 (1330-1680)	103 (46.4 - 161.0)	13.1 (5.92 - 20.8)
**Black**	58900 (58000-59800)	37100 (36100-38100)	5350 (4670-6050)	106 (48.1 - 166.0)	9.63 (4.31 - 15.4)
**Hispanic**	61200 (60600-61800)	43200 (42500-43900)	7500 (6700-8300)	101 (45.8 - 158.0)	12.4 (5.55 - 19.6)
**White**	63700 (63000-64400)	37100 (36400-37900)	8720 (8110-9350)	105 (47.3 - 163.0)	14.3 (6.48 - 22.5)

VI. eReferences

1. Prener CG, Fox B. Creating open source composite geocoders: Pitfalls and opportunities. *Trans GIS*. 2021;25(4):1868-1887. doi:10.1111/tgis.12741
2. US Census Bureau. American Community Survey. Published online 2023. https://www.census.gov/programs-surveys/acs
3. Azur MJ, Stuart EA, Frangakis C, Leaf PJ. Multiple Imputation by Chained Equations: What is it and how does it work? *Int J Methods Psychiatr Res*. 2011;20(1):40-49. doi:10.1002/mpr.329
4. HCS Resources. Accessed December 27, 2023. https://www.chicago.gov/content/city/en/depts/cdph/supp_info/healthy-communities/resources.html
5. City of Chicago Data Portal. Chicago Building Footprints. Published online 2023. https://data.cityofchicago.org/Buildings/Building-Footprints-current-/hz9b-7nh8
6. Chicago Department of Public Health, UIC Phame Center, Metopio. Chicago Health Atlas. Published online 2023. https://chicagohealthatlas.org/
7. Ke G, Meng Q, Finley T, et al. LightGBM: A Highly Efficient Gradient Boosting Decision Tree. In: *Advances in Neural Information Processing Systems*. Vol 30. Curran Associates, Inc.; 2017. Accessed July 13, 2023. https://proceedings.neurips.cc/paper/2017/hash/6449f44a102fde848669bdd9eb6b76fa-Abstract.html
8. Breiman L. Random Forests. *Mach Learn*. 2001;45(1):5-32. doi:10.1023/A:1010933404324
9. Friedman J, Hastie T, Tibshirani R. Regularization Paths for Generalized Linear Models via Coordinate Descent. *J Stat Softw*. 2010;33(1):1-22.
10. Max Kuhn. tune: Tidy Tuning tools. Published online 2023. https://tune.tidymodels.org/
11. Cribari-Neto F, Zeileis A. Beta Regression in R. *J Stat Softw*. 2010;34:1-24. doi:10.18637/jss.v034.i02
12. Forman G. Quantifying counts and costs via classification. *Data Min Knowl Discov*. 2008;17(2):164-206. doi:10.1007/s10618-008-0097-y
13. Bella A, Ferri C, Hernández-Orallo J, Ramírez-Quintana MJ. Quantification via Probability Estimators. In: *2010 IEEE International Conference on Data Mining*. ; 2010:737-742. doi:10.1109/ICDM.2010.75
14. Fiksel J, Datta A, Amouzou A, Zeger S. Generalized Bayes Quantification Learning under Dataset Shift. *J Am Stat Assoc*. 2022;117(540):2163-2181. doi:10.1080/01621459.2021.1909599
