# Supplementary material for: Estimated Childhood Lead Exposure From Drinking Water in Chicago
Source: JAMA Pediatr. 2024 Mar 18;178(5):473–9. doi: 10.1001/jamapediatrics.2024.0133 (PMC10949143; doi:10.1001/jamapediatrics.2024.0133)
Supplement: Supplement 2. — Data Sharing Statement [file jamapediatr-e240133-s002.pdf]

Data Sharing Statement

Huynh. Estimated Childhood Lead Exposure From Drinking Water in Chicago. *JAMA Pediatr*. Published March 18, 2024. doi:10.1001/jamapediatrics.2024.0133

Data
Data available: Yes
Data types: Data (not involving human participants)
How to access data: All data used are publicly available, except for responses from the Healthy Chicago Survey. Aggregate data and intermediate processed datasets will be available upon request at bhuynh@jhu.edu.
When available: With publication

Supporting Documents
Document types: Statistical/analytic code
How to access documents: Code will be made available in a github repository at https://github.com/benhuynh/chicagoLeadExposure
When available: With publication

Additional Information
Who can access the data: Code will be made freely available
Types of analyses: Any purpose
Mechanisms of data availability: All data except data from the Healthy Chicago Survey will be freely available, as they are publicly available and do not contain confidential information. Data from the Healthy Chicago Survey cannot be shared.
